# The importance of deep speech phenotyping for neurodevelopmental and genetic disorders: a conceptual review

**DOI:** 10.1186/s11689-022-09443-z

**Published:** 2022-06-11

**Authors:** Karen V. Chenausky, Helen Tager-Flusberg

**Affiliations:** 1 Speech in Autism and Neurodevelopmental Disorders Lab, Massachusetts General Hospital Institute of Health Professions, 36 1st Avenue, Boston, MA 02129 USA; 2 Department of Neurology, Harvard Medical School, Boston, USA; 3 Department of Psychological and Brain Sciences, Boston University, Boston, USA

**Keywords:** Speech sound disorders, Childhood apraxia of speech, Language, Phenotype, Motor speech disorders

## Abstract

**Background:**

Speech is the most common modality through which language is communicated, and delayed, disordered, or absent speech production is a hallmark of many neurodevelopmental and genetic disorders. Yet, speech is not often carefully phenotyped in neurodevelopmental disorders. In this paper, we argue that such deep phenotyping, defined as phenotyping that is specific to speech production and not conflated with language or cognitive ability, is vital if we are to understand how genetic variations affect the brain regions that are associated with spoken language.

Speech is distinct from language, though the two are related behaviorally and share neural substrates. We present a brief taxonomy of developmental speech production disorders, with particular emphasis on the motor speech disorders childhood apraxia of speech (a disorder of motor planning) and childhood dysarthria (a set of disorders of motor execution). We review the history of discoveries concerning the KE family, in whom a hereditary form of communication impairment was identified as childhood apraxia of speech and linked to dysfunction in the *FOXP2* gene. The story demonstrates how instrumental deep phenotyping of speech production was in this seminal discovery in the genetics of speech and language. There is considerable overlap between the neural substrates associated with speech production and with *FOXP2* expression, suggesting that further genes associated with speech dysfunction will also be expressed in similar brain regions. We then show how a biologically accurate computational model of speech production, in combination with detailed information about speech production in children with developmental disorders, can generate testable hypotheses about the nature, genetics, and neurology of speech disorders.

**Conclusions:**

Though speech and language are distinct, specific types of developmental speech disorder are associated with far-reaching effects on verbal communication in children with neurodevelopmental disorders. Therefore, detailed speech phenotyping, in collaboration with experts on pediatric speech development and disorders, can lead us to a new generation of discoveries about how speech development is affected in genetic disorders.

## Background

Spoken language is a uniquely human skill that, when disordered, is often a salient presenting symptom of a neurodevelopmental or genetic disorder (NDD) [71]. For example, of 302 genetic syndromes described in Shprintzen [73], speech was affected at least some of the time in 235 (78%) of them. There has been tremendous growth in research on NDDs since Shprintzen’s book was published. Many newly discovered genetic syndromes associated with speech deficits, resulting from different types of mutations, have been and will continue to be identified at a rapid rate (e.g., [8, 40, 62, 103]). At the same time, the genetic influences on common disorders related to language and literacy, including autism (ASD), developmental language disorder, and dyslexia, are also advancing (for reviews, see [22, 34, 38]). It is clear that most genetic mutations associated with these disorders have widespread effects on the developing brain and influence not only language but also cognitive abilities more broadly. However, phenotyping of the range of speech and language profiles in research on specific disorders varies widely. Many studies simply describe affected children as having “intellectual disability” or “speech delay”, even at times seeming to use these terms interchangeably (e.g., [19, 83]). Others employ more specific measures of IQ (intelligence quotient), language, and speech to produce more detailed and accurate phenotypes (e.g., [82]). But because IQ, language, and speech are separable (though interacting) cognitive domains with distinct (though overlapping) neural substrates, in order to understand the full range of effects of specific genetic mutations, it is necessary to evaluate them all using psychometrically sound measures.

In this review, we focus on the key communication subcomponent of *speech production*. Our overall argument is that it is critical for studies aiming to characterize language and communication phenotypes in NDDs to collect measures of speech production, in part because speech is the most common modality of expressing language and because early vocalizations are highly predictive of later aspects of language such as vocabulary size (e.g., [101]). To set the stage, we begin by distinguishing speech and language—an important distinction, because the neural substrates associated with speech are different from those traditionally related to language [39]. Thus, speech development is likely to be under the control of some distinct genetic factors. Next, because impaired speech is so common in NDDs, we briefly review a taxonomy of developmental speech disorders.

After establishing this background, we then review the story of the KE family and the discovery of the *FOXP2* gene. There, careful phenotyping of speech production was at least as significant as that of language ability in characterizing the family’s overall communication impairment. Their story and that of *FOXP2* clearly illustrate the importance of deep speech phenotyping in NDDs, which we define as characterizing behaviors that are specific to speech production, elucidating aspects of typical or disordered speech development, and avoiding conflation of speech with language or cognitive ability. Transitioning to the present day, we then show how a recent computational model of speech production, undergirded by careful speech phenotyping, can accelerate research in developmental speech disorders. We finish by providing guidance on protocols for speech phenotyping.

Throughout, we point out both the work that has already been done to understand how speech development is altered in NDDs, as well as the many areas that still require research. This underscores our theme that careful, thorough descriptions of speech performance are required in order to formulate testable hypotheses about how genes related to language and communication disorders affect brain development and, therefore, speech production. Because neural structures are determined by both genetics and behavior, computational models of speech production have the power to provide explanations at multiple levels and, eventually, link genetics to behavior via neurology and computation. The utility of explanatory models of genetic function, neural development, and speech production fundamentally depends on accurate, high-quality phenotyping data.

## Speech vs. language

An important first step is to distinguish speech from language. There is no hard-and-fast definition of either term, but the two can be described as follows: language is an abstract, rule-governed, generative system of symbols that humans use to express ideas. Aspects of language include semantics (meaning), morphosyntax (word and sentence structure), pragmatics (the social use of language), and phonology (pronunciation rules). Language can be expressed through a variety of modalities. These include manual signs as in, for example, American Sign Language; written characters like those you are reading now; and speech. Speech is the spoken mode of language and involves both the auditory and oral-motor systems. It is created by laryngeal vibration and modification of the resulting sound by movement of articulators such as the lips, tongue, and velum [86].

Speech is also the overwhelmingly most common mode of expressing language and thus garners more research emphasis than sign or text. However, in focusing on speech, we do not intend to minimize the importance of these other modalities. Sign and text can tell us much about the human capacity for language and communication and are sorely under-researched, especially as communication modes for people who do not use speech.

Another caveat about speech research is that the bulk of previous work has concerned adults, whether typical speakers (e.g., [86, 94]) or speakers with disorders such as Parkinson’s disease, stroke, or amyotrophic lateral sclerosis (e.g., [25]). There is a growing body of literature on speech production in typically developing children [18, 51], which informs our focus here on the developmental speech disorders that are so common in children with NDDs. Because much less research has focused on the speech of children with NDDs than on other types of speech disorders, however, we should not expect that all conclusions based on these other populations will be borne out in the speech of children with NDDs.

It is likely that models of speech development based on adult neurobiology will not suffice to explain typical or atypical developmental courses, and our call for deep phenotyping of speech development in NDDs is aimed specifically at collecting the kind of detailed information that is required to construct developmental models that accurately reflect the range of speech phenotypes that exist. In addition to the information itself, models such as the Interactive Specialization framework proposed by Mark Johnson [47] will be needed. This model takes behavior-dependent interaction between different brain networks into account to explain how changes in the activity of these networks is associated with the appearance of new skills. It posits that interactions between genes, brain structures, and behaviors are dynamic and bidirectional and that the brain is both self-organizing and activity-dependent. In Johnson’s model, the same behavior may be supported by different neural substrates at different times during development, with a general developmental movement toward increasing specialization and localization of brain regions serving a specific behavior over time, supported by neural process such as synaptic pruning and the plastic reorganization of specific networks and their connections to other networks.

In a related vein, Kent [49, 50] proposed that a series of developmental functional modules for speech can explain the different vocalization types that typically developing infants, toddlers, and children produce at different ages. These modules, which cover the vocal production components of respiratory, laryngeal, mandibular, lingual, labial, velopharyngeal, and pharyngeolaryngeal performance, are based on a series of biological developmental processes that bring about a significant remodeling of the anatomical systems serving speech. Consistent with the Interactive Specialization Framework, these modules rely on computational principles of movement variability, self-organization, and synergy with other modules to achieve stable performance over the course of childhood in typical development. Clearly, more detailed research, including longitudinal studies, is needed to understand how behavior, experience, and neural structures interact over the course of development to produce the range of phenotypes we see across NDDs, especially in cases of atypical development.

## Developmental speech disorders

Becoming a competent user of speech requires the intent to communicate, the cognitive ability to formulate a meaningful message, and the oromotor skills to produce the sounds that communicate that message to others. While the great majority of children develop speech without difficulty, approximately 9% of children in the USA have noticeable speech disorders (NIDCD [66], https://www.nidcd.nih.gov/health/statistics/quick-statistics-voice-speech-language). As mentioned, speech disorders are much more common in NDDs than in the general population and can be caused by a variety of factors. The degree of impairment can range from mild to very severe; in some cases, speech is altogether absent. Speech performance has downstream influences on other areas of development, particularly language and literacy (e.g., [101]).

**Fig. 1:**
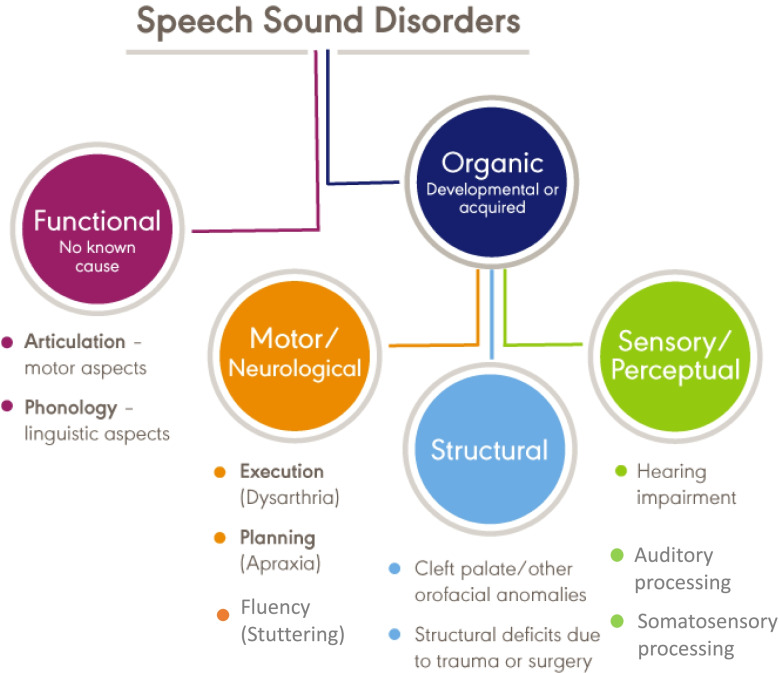
Taxonomy of Speech Disorders (adapted from https://www.asha.org/practice-portal/clinical-topics/articulation-and-phonology/). Note: “Motor Speech Disorders-Not Otherwise Specified” does not appear on this diagram

The American Speech-Language-Hearing Association (ASHA) classifies developmental speech disorders into two major categories, with subdivisions under each (Fig. 1 [2]). Functional, or idiopathic, speech disorders contrast with organic disorders. The former include disorders of articulation, which are distortion or substitution errors that affect individual phonemes—an example might be a misarticulation of “r” that makes it sound like “w” (e.g., “wabbit” for “rabbit”). Another type of functional speech disorder is phonological disorder, which involves predictable, rule-based errors that affect more than one speech sound, such as final consonant deletion.

Organic speech disorders can be structural, sensory/perceptual, or motor/neurological in origin. Structural speech disorders result from congenital orofacial anomalies such as cleft palate. Sensory/perceptual speech disorders arise as a consequence of conditions like hearing impairment, auditory processing disorder, or somatosensory disturbances. Finally, motor/neurological speech disorders include stuttering (a fluency disorder), childhood apraxia of speech (a motor planning disorder), and childhood dysarthria (a set of motor execution disorders). The category “motor speech disorder-not otherwise specified” (MSD-NOS, not shown in Fig. 1) acknowledges that this list may not be exhaustive.

Comorbid disorders of motor planning are highly relevant to communication development in NDDs, since they are more closely linked with overall language and literacy development than structural disorders, dysarthria, or stuttering and put children at risk for later language and literacy challenges even in those cases where the initial disorder has resolved [46, 56, 65, 85, 90]. Recent comorbidity studies have shown that children with disorders of motor planning plus other NDDs experience more severe communication profiles than children without such comorbidity [10, 14, 16, 43]. Research on how comorbid communication disorders affect each other is in its infancy but promises to reveal much about the underlying genetic, behavioral, and neural differences associated with these disorders [69].

Below, we discuss in more detail two motor speech disorders, childhood apraxia of speech and childhood dysarthria, both of which are common comorbidities in different NDDs.

## Childhood apraxia of speech

Childhood apraxia of speech (CAS) deserves particular attention, both because it is persistent and often severe and because of its association with members of the KE family, whom we will discuss below. CAS is defined as “a neurological speech sound disorder in which the precision and consistency of movements underlying speech are impaired in the absence of neuromuscular deficits” ([1], p. 2). It is diagnosed using a motor speech examination, which allows a clinician to observe a child’s speech performance across a set of tasks and stimuli that vary systematically in complexity and length. A number of individual signs may be noted [11, 45, 79, 80, 89], which together indicate (a) inconsistent errors on repeated productions of syllables or words; (b) lengthened and disrupted coarticulatory transitions between phonemes; and (c) disordered prosody, manifest in incorrect application of stress and inappropriate pausing within and between words [1, 11]. As a sole diagnosis, CAS has been estimated to affect 1–2 children per thousand [74]. Like stuttering, CAS is associated primarily with cortical, rather than subcortical, differences [54, 58].

## Childhood dysarthria

Childhood dysarthria is also common in NDDs. Classification of childhood dysarthria types is based on models of adult dysarthria, in which observable symptoms are linked to different disorders of the cranial nerves, neuromuscular junctions, cerebellum, basal ganglia, and corticobulbar tracts while the cortical substrates of speech are largely intact [20, 21, 25]. We describe the main types of dysarthria, along with their neurological correlates, as they have been characterized in adults; however, it is unknown how well this taxonomy applies to childhood dysarthria or how dysarthrias associated with developmental disorders evolve over the course of development.

*Flaccid dysarthria* is associated with lower motor neuron or neuromuscular junction dysfunction and is characterized by weakness, low muscle tone, altered laryngeal vibratory characteristics such as diplophonia, and continuous hypernasality. Flaccid dysarthria is commonly found in disorders such as Prader-Willi syndrome and myasthenia gravis, among others. *Spastic dysarthria* is due to disorders affecting upper motor neurons and is characterized by slow, effortful speech; tense or harsh voice; and pitch breaks. Hereditary spastic paraplegias and cerebral palsy are often associated with spastic dysarthria in children. *Ataxic dysarthria* is associated with disorders of the cerebellum, such as spinocerebellar and Friedreich ataxia, and is characterized by irregular interruptions in speech, variable errors, and dysmetric speech movements. *Hypokinetic dysarthria* is most commonly associated with Parkinsonian syndromes that affect the substantia nigra of the basal ganglia. It is characterized by a reduction in the intensity and range of motion of speech, giving the impression of mumbled, faint, and speeded-up speech. *Hyperkinetic dysarthria* is related to disorders of the basal ganglia pathway that affect the caudate and/or putamen, such as spasmodic dysphonia. It is characterized by tense speech; rapid fluctuations in volume; and sudden inhalations, exhalations, or speech interruptions. Finally, *mixed dysarthrias* incorporate features of more than one of the other forms of dysarthria. NDDs such as cerebral palsy or Down syndrome (DS) may be associated with different types of dysarthria (e.g., flaccid and/or ataxic) at the same time or at different points in development [52].

The careful reader will have noticed that many of the characteristics of different dysarthrias bear a similarity to the core impairments in CAS. For example, “lengthened and disrupted coarticulatory transitions between phonemes”, a characteristic of CAS, could be caused by “irregular interruptions in speech”, a characteristic of ataxic dysarthria. For this reason, differential diagnosis is challenging and requires the collaboration of a speech-language pathologist with specific expertise in pediatric motor speech disorders, along with the use of valid assessments. Complicating the situation, CAS and childhood dysarthria can of course co-occur. For example, a recent study of conversational speech samples from adolescents with DS showed that over 97% met criteria for motor speech disorders including CAS, childhood dysarthria, and speech motor delay [102]. While precise figures documenting the co-occurrence of CAS and childhood dysarthria are lacking, Shriberg et al. [81] provided estimates of 4.9% of children with one of eight complex neurodevelopmental disorders meeting criteria for concurrent CAS and childhood dysarthria. A recent retrospective study of comorbidity in 375 children with CAS showed that 6.7% (25) also carried diagnoses of dysarthria [10, 14, 16].

## The KE family

We move now to a brief overview of the history of discoveries about the KE family. The story illustrates how deep speech phenotyping in individuals with NDDs not only elucidated the links between genetic mutations and behavioral phenotypes, but also shed light on our understanding of the nature of their communication impairment as well as its neural and genetic bases.

Hurst et al. [41] were the first to report on six members of an extended family who had been referred to a genetic clinic because of their severely impaired verbal communication. The disorder was referred to as “a severe form of developmental verbal apraxia” but was characterized by a variety of abnormal features relating to both speech and language. For example, case study participants were described as having unintelligible speech, but also as showing word-finding problems, using telegraphic speech (speaking in short sentences without morphological inflections), and having below-average language comprehension. The disorder was complex in nature and varied both in severity and in its manifestation across family members.

Soon after, three competing viewpoints emerged. Gopnik and colleagues [32, 33] conceived of the family’s disorder as being mainly one of “dysphasia” – in other words, language-based – after having administered a set of tests for aphasia and examining writing and conversational samples. Underlying the various manifestations of the disorder, in their view, was an inability to “infer general rules about… grammatical features” [32] or “construct an underlying grammar for abstract morphemes like number and tense” [33]. In contrast, Fletcher [27] noted that, while affected family members scored, on average, much lower than unaffected members on tests of grammatical features such as pluralization or past tense, they did sometimes produce the correct form, which could be the result of severe phonological disorder. In other words, what appeared to be a grammatical impairment might actually have been due to a phonological process like final-consonant deletion. Finally, Vargha-Khadem and Passingham [97] pointed out that affected family members’ ability to repeat individual speech sounds, words, nonwords, and sentences were all impaired, as well as their ability to correctly name or identify objects.

The conflicting hypotheses were resolved by further and deeper phenotyping. Using a larger, more detailed set of tasks and stimuli, Vargha-Khadem et al. [98] showed that affected family members made over-regularization errors on past tense (saying “runned” for “ran”, for example), showing that they did possess some grammatical knowledge and making the hypothesis of a profound grammatical deficit less tenable. In addition, family members’ performance on tests of oral and facial movement (including tasks like “click your tongue” or “close your left eye”) showed that, while affected and non-affected family members performed equally well when asked to perform one motion at a time, affected family members were significantly less able to correctly produce sequences of these movements. Following up on these findings, Vargha-Khadem et al. [99] showed that all affected family members (but no unaffected family members) were impaired on three types of task: word repetition, nonword repetition, and performing sequences of orofacial movements. Thus, they concluded, the core impairment in the disorder was not language impairment or phonological disorder but one of verbal dyspraxia—what we now term CAS. Whether affected family members’ associated deficits, such as grammatical, semantic, and nonverbal IQ impairments, represented consequences of CAS or additional core deficits that only affected a smaller number of family members was left an open question. Other studies have documented that language impairment is a common comorbidity in children with CAS [10, 14, 16, 44, 65, 92], though the connection is still poorly understood.

In parallel with the above investigations, other studies sought the genetic basis of the KE family’s disorder. Lai et al. [55] found that genetic differences in a region of chromosome 7 correlated with KE family members’ affectation status. These researchers also coincidentally encountered an unrelated patient whose speech and language impairments closely resembled that of affected KE family members. Part of this patient’s chromosome 7 had broken off and reattached to part of chromosome 5. The break occurred in the same region identified in the KE family correlation analysis. Further investigation revealed that in both cases a gene coding for forkhead transcription factors—later named *FOXP2—*was affected in both the KE family and the unrelated patient. For the first time, a clear link between spoken language impairment and a genetic mutation had been identified.

## Genes associated with motor speech disorders in NDDs

Since the seminal work on *FOXP2*, multiple studies have found that speech disorders are especially frequent in syndromes associated with specific genomic events at the chromosomal, copy number variant (CNV), or single gene level [6, 26, 61, 63, 64, 77, 78, 81]. With the advent of the human genome project and the rapid growth in technology for identifying mutations, many new rare NDDs have been recognized in which motor speech disorders are quite prevalent [38]. As an aside, it is interesting to note that several of these mutations were initially discovered in cohorts of children ascertained for ASD, even though until recently ASD was not believed to include co-occurring motor speech disorders (c.f [77, 78]., but also [13]). In Table 1, we provide examples of specific genes or CNVs that have been associated with CAS, drawing primarily on the recent review by Guerra and Cacabelos [38] and a detailed genomic investigation of a cohort of probands with CAS [40].

**Table 1:** Examples of specific genes or CNVs that have been associated with CAS

Chromosome	Locus	Gene	Citation
1	1p36.33	*GNB1*	[40]
	1q21.3	*POGZ*	
2	2q25	*ZNF142*	[40]
3	3p13	*FOXP1*	[38]
	3q29	*ATP13A4*	
5	5p14.3	*CHD18*	[38]
	5p15.1	*MY010*	
	5q13.2	*NIPBL*	
6	6p22.3	*KIAA0319*	[38]
7	7p11.2	*FLCN*	[38]
	7p14.1	*CDK13*	[40]
	7q31.1	*FOXP2*	[38]
	7q35-q36	*CATNAP2*	[38]
8	8p11.21	*KAT6A*	[38]
	8q21.13	*ZFHX4*	
9	9q34.12	*SETX*	[38]
	9q34.2	*WDR5*	
10	10q26.2	*EBF3*	[40]
11	11p11.2	*SMCR8*	[38]
12	12p13.33	*ELKS*	[38]
15	15q14	*MEIS2*	[40]
	15q25.1	*ZGRF1*	[38]
16	16p11.2	*SETD1A*	[38]
	16p13.2	*GRIN2A*	[95]
	16q13	*GNAO1*	[40]
17	17p12-p11	*NCOR1*	[38]
	17p13.1	*CHD3*	
	17q11.2	*NEK8*	
	17q21.2	*CATNAP1*	
	17q21.31	*UPF2*	[40]
18	18p11.22	*ANKRD12*	[38]
	18q12.3	*SETBP1*	[40]
22	22q13.1	*TNRC6B*	[38]
	Xp11.4	*DDX3X*	[40]

Importantly, many published case study descriptions of NDDs, which are identified on the basis of a genomic event (for a review, see [59]), do not mention the presence of motor speech disorders because they fail to include in the clinical team speech pathologists with the appropriate diagnostic expertise. Instead, most of what we know comes from studies that ascertained cases with motor speech deficits and then carried out genetic testing or were designed to investigate speech in specific genetic cohorts.

## Neural substrates associated with speech production

So far, we have shown that motor speech disorders are highly prevalent in NDDs and documented some of the specific genes and CNVs associated with NDD/motor speech disorder comorbidity. But genes are related to behavioral phenotypes via the brain. Therefore, a more complete picture of the significance of speech disorders will result from considering the neural substrates for speech and language. Neural structures associated with speech production are largely (though not exclusively, as we shall see below) located near the Rolandic cortex, the region including and adjacent to the central sulcus [24, 37, 39]. Primary motor cortex, especially the inferior portion of the motor strip just anterior to the central sulcus, generates the motor commands that are sent to the articulators via cranial nerves VII, IX, X, and XII [53]. Primary somatosensory cortex, just posterior to the central sulcus, is responsible for somatosensory feedback-based speech control via cranial nerves V, IX, and X [67, 68]. Primary auditory cortex not only plays a role in perception of heard speech [28], but also in auditory feedback control of one’s own speech via cranial nerve VIII [93]. Auditory feedback plays an especially important role in speech acquisition because it is crucial to establishing the mappings between articulator movement and acoustic output [7, 72].

Somatosensory and motor representations are integrated in the ventral portion of the Rolandic cortex. Neural representations of the speech articulators are arranged dorsally to ventrally, with respiratory structures being represented most dorsally and representations of the larynx, lips, jaw and tongue appearing increasingly ventrally ([17, 48]. Like the motor and somatosensory regions, these representations overlap, which allows for inter-coordination between articulators during speech. Finally, left frontal cortex carries out the highest levels of speech motor planning [37].

The two main white-matter tracts known to be involved in speech production are the arcuate fasciculus (AF) and the frontal aslant tract (FAT). The AF is a horizontal pathway connecting temporal cortex and Broca’s area [70] and is considered responsible for linking auditory representations of speech sounds with the movements required to produce them. The FAT is a vertical pathway that extends from the superior frontal gyrus to the posterior portion of the supplemental motor area (SMA) and the pre-SMA, ending in the inferior frontal gyrus (IFG) [9]. It plays a role in initiating speech and has found to be abnormal in adults with developmental stuttering compared to typically developing controls [54].

Other white-matter tracts that have been associated with speech production are the superior longitudinal fasciculus (SLF [3, 23, 29, 30];) and the corpus callosum [5, 60]. However, the research supporting these tracts’ involvement in speech production is less clear-cut and, in the case of the corpus callosum, illustrative of the phenotyping issues we raise here. In one study, Bartha-Doering et al. [5] examined the relationship of a series of language measures and measures of corpus callosum volume along its full extent in a group of 38 typically developing children. Among the language measures they used was a test of expressive vocabulary, where children name pictures and which is scored according to semantic criteria (i.e., whether the child refers to the picture with the correct word). They found that the posterior subsection volume of the corpus callosum was significantly positively related to expressive vocabulary score. By contrast, Luders et al. [60] compared children with functional speech sound disorders to typically developing controls. Children were assessed with standardized tests of articulation and of language, and a conversational speech sample was also collected. The articulation test, also a picture-naming task, was scored according to whether children could *pronounce* the name of the picture correctly. If they did not spontaneously produce the correct target word, children were asked to repeat it after the examiner. Both the articulation test responses and the speech sample were coded for speech sound errors. Children with speech sound disorders had corpus callosa that were significantly thinner than those of control children, especially in the anterior third of the tract, which is closely connected to frontal, premotor, and supplemental motor areas of cortex.

It is not possible to know whether the discrepant results from these two studies are due to the tests administered, the participants, or both. What we would like to point out is the difference between tests of language, such as an expressive vocabulary test, and tests of speech, such as an articulation test; and to underscore that even when the task put to the participants is almost identical in both cases (picture naming), how those tests are scored or coded is what determines whether the results pertain to language or speech.

## Neural substrates associated with FOXP2 expression

The finding that orofacial and speech apraxia are core deficits in the KE family allowed Vargha-Khadem et al. [99] to formulate hypotheses about the neural basis of these deficits, which they tested using voxel-based morphology (VBM) to identify regions of gray or white matter that differed between affected family members and controls in an effort to understand the neural differences between affected and unaffected family members that would result in the behavioral phenotypes they saw. On the basis of their behavioral data, Vargha-Khadem et al. [99] hypothesized that brain regions related to the motor system would be involved bilaterally in affected family members. Results confirmed these hypotheses: affected family members showed functional abnormalities in pre-supplementary motor area (SMA), Broca’s area and its right-hemisphere homolog, and significantly smaller caudate nuclei. These findings were corroborated by two additional studies [57, 99]. In a PET study, affected family members showed overactivation in the caudate, premotor cortex (with a ventral extension into Broca’s area), and ventral prefrontal cortex; and underactivation of SMA, pre-SMA, and cingulate cortex during speech tasks. Volumetric analyses showed that affected family members’ caudate nuclei were approximately 75% of the size of those in unaffected family members and age-matched control participants [99]. Later fMRI studies involving speech tasks showed significantly less activation in affected family members in Broca’s area, its right-hemisphere homolog, and in the putamen [57].

Abnormalities in Broca’s area and premotor cortex in affected KE family members are thus consistent with difficulties linking auditory representations of speech sounds to articulatory movements, an aspect of motor planning that is disordered in CAS. Recent work supporting this finding has identified AF abnormalities in children with CAS [58]. Other research has shown that minimally verbal children with ASD, a population in which CAS is common [13], have smaller AF volume in the left hemisphere and reversed AF laterality relative to typical controls [100]. Structural abnormalities of the left AF have also been shown to be inversely related to the amount of improvement in speech production that minimally verbal children with ASD and CAS experience after therapy [15], and integrity of the right FAT was related to the degree of syllable insertion errors in the same group of children.

**Fig. 2:**
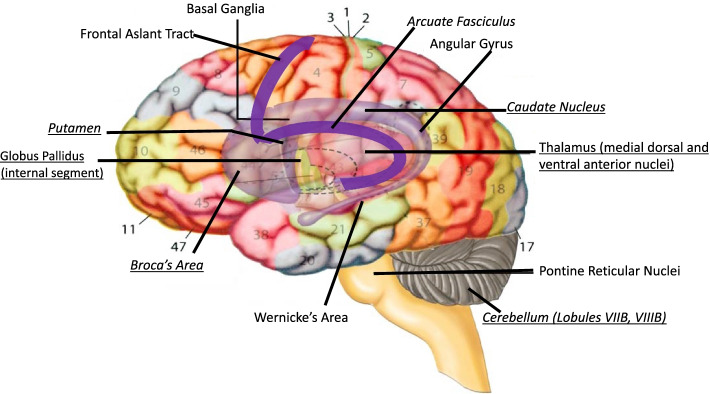
Cortical and subcortical areas related to spoken language. Underlined regions express FOXP2. Regions in *italics* are structurally or functionally abnormal in KE family members

In the almost 20 years since the groundbreaking discovery of *FOXP2*, its role in human brain development has been investigated more thoroughly. It is expressed in many brain areas: in sensory nuclei, various locations in cortex, and in specific subdivisions of motor regions [96] which led Vargha-Khadem and her colleagues to propose a spoken-language circuit that is dependent on *FOXP2*. The structure of this circuit and its relevance to the speech deficits experienced by KE family members dovetails with other attempts to link what is known about the neural basis of speech to behavioral aspects of speech production. Figure 2 illustrates many of the cortical and subcortical regions involved in spoken language production, highlighting areas that are also abnormal in affected KE family members and areas in which *FOXP2* is expressed.

In addition to studies directly linking imaging and behavioral results in the same individuals, other research has combined what is known from studies of typical and disordered function to construct computational models of speech production. In turn, these models can be used to formulate testable hypotheses about which brain regions are expected to be affected in children with different speech sound disorders. Below, we discuss one such model, the Directions into Velocities of Articulators (DIVA) model [35–37].

## The DIVA model of speech production

DIVA is a biologically accurate neural network that models the feedforward and feedback control loops involved in both developing and mature speech production. Its developmental model includes three stages, the first of which approximates infant babbling. In this stage, semi-random motor commands enable the model to learn the mapping between oral movements and their acoustic and somatosensory consequences. The model also acquires the mappings that translate sensory errors into corrective motor commands. These mappings are the core of DIVA’s auditory and somatosensory feedback control systems.

In the second developmental stage, DIVA “learns” to imitate targets containing phonemes from its “native” language. DIVA’s initial attempts to produce the phonemes rely heavily on auditory feedback control, but motor commands are updated after each attempt and stored in synaptic weights that encode feedforward motor programs for the phonemes. In this way, pronunciation improves over repeated attempts, eventually allowing DIVA to produce phonemes with minimal contribution from the feedback system: the learned feedforward commands are then sufficient for error-free production. This is the mature stage of development, in which DIVA relies on the feedforward commands it has refined in order to consistently produce correct utterances.

**Fig. 3:**
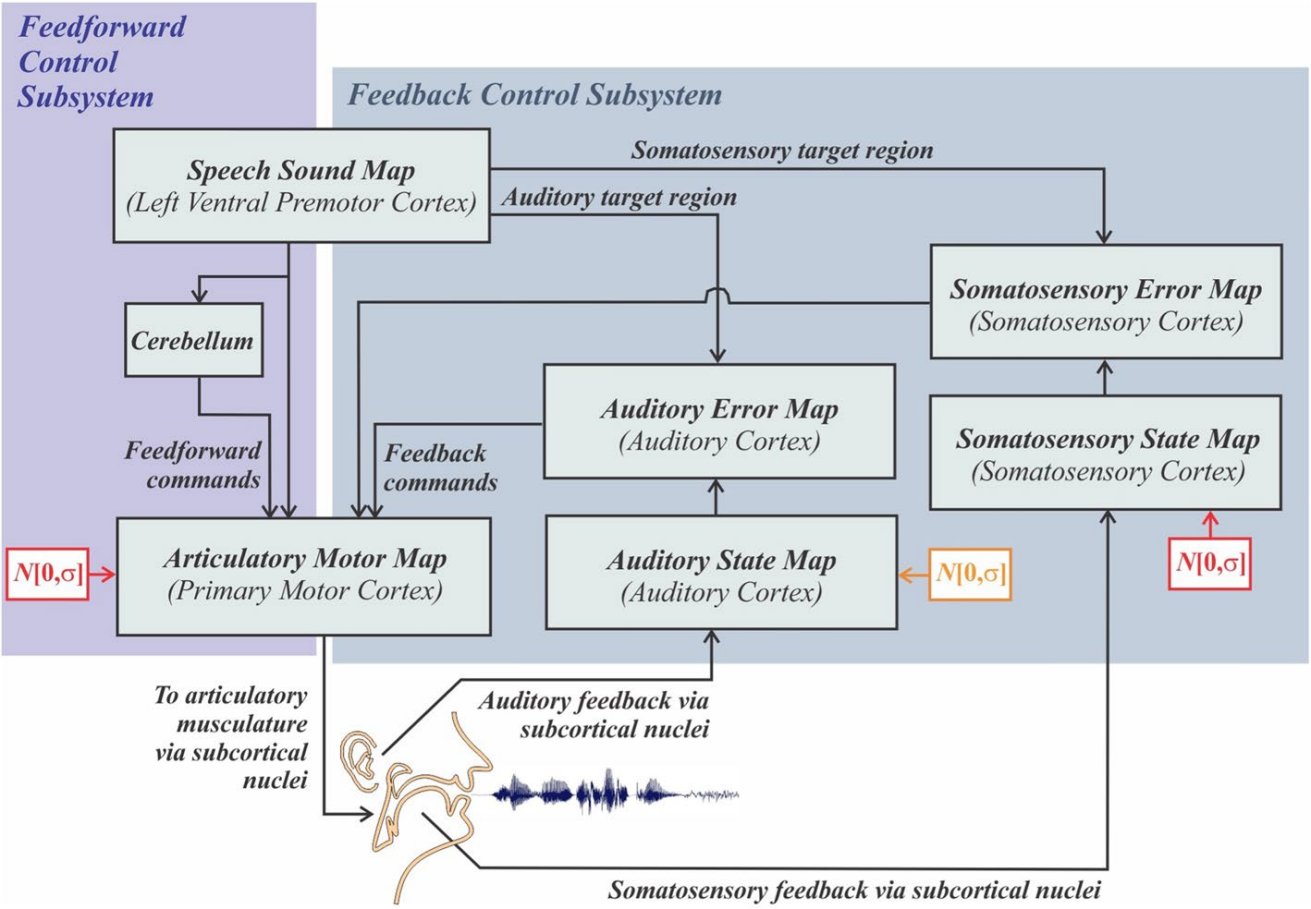
Illustration of the DIVA model and its neural correlates

In addition to modeling typical speech development, DIVA can also model developmental disorders of speech. Terband et al. [91] used DIVA to simulate the effect of two developmental disorders. First, DIVA was given a “motor processing disorder” by adding Gaussian noise to the articulatory velocity/position and somatosensory state maps during its developmental stage (indicated by the red boxes in Fig. 3). The result was speech that was highly distorted (i.e., very different from the target) and highly variable (repeated attempts at the same target resulted in widely different output). Distortion and variability of the model’s speech arose from instability in the motor and sensory commands required to accurately position the articulators, as well as from the model’s attempt to use its auditory feedback to refine its acoustic output and arrive at the correct sound. Next, DIVA was also given an “auditory processing disorder” by adding noise to the auditory state map (indicated by the orange box in Fig. 3). This had the effect of removing DIVA’s ability to use auditory feedback, as well as making phonemes sound more alike to the model. The result in this case was that all the model could produce was “uh”. Extending this work, Chenausky et al. [12] tested these predictions on the speech of a group of 38 minimally verbal children with ASD, some of whom had comorbid CAS. Using both perceptual and acoustic analysis methods, two groups emerged. Twenty-seven children showed distorted, variable speech, corresponding to the “motor processing disorder” version of DIVA. The remaining 11 showed speech that was limited to versions of “muh” and “buh”, more closely resembling the “motor plus auditory processing disorder” version of DIVA.

Taken together, these simulation studies leverage the similarities between the DIVA model and the *FOXP2*-dependent speech circuit proposed by Vargha-Khadem et al. [96]. There is a high degree of overlap between the regions in which *FOXP2* is expressed and those involved in the feedforward control system for speech. The ventral premotor cortex, portions of the cerebellum, the ventral lateral thalamic nucleus, ventral motor cortex, supplementary motor area, putamen, globus pallidus and portions of the substantia nigra, and the ventral anterior thalamic nucleus all appear in both circuits. The DIVA model thus informs us as to what computational aspects of speech are impaired in affected KE family members. Specifically, it offers independent confirmation that affected KE family members experience difficulty with feedforward speech control processes—that is, they have difficulty in knowing how to move their mouths in order to produce the correct sound sequences fluently and intelligibly.

## Conclusions and recommendations for future work

Together, all of these lines of research—speech development, developmental motor speech disorders, familial motor speech impairments, the genetic bases of motor speech impairment, imaging studies documenting the specific neural impairments associated with motor speech impairment, and computational modeling of motor speech impairment—tell a complex and informative story about how speech production can be affected in individuals with NDDs. Implicit in the timeline of these discoveries is that they were driven in large part by careful observation of anomalous spoken-language behavior and a desire to understand its neural and genetic origins. In other words, deep speech phenotyping can be a major driver of the discoveries like the ones we have discussed here.

The story of the KE family, viewed in the context of what is now known about the neural bases of speech production and the high prevalence of motor speech disorders in NDDs, makes several other points as well. First, the neural regions associated with speech production are separate from those associated with language per se and are ones in which *FOXP2* expression is high. But because the neural regions for speech do interact with those for other aspects of language, it is possible that disruptions to them during the developmental period also affect language development. Specifically, neural developmental differences in the regions involved in creating the forward model of speech production may result not only in problems sequencing speech movements, but also in problems sequencing nonspeech oral movements and in sequencing words within sentences. The more general question of how CAS is related to language and literacy impairments is an open one, deserving of more research.

The discovery that CAS is the core impairment in the affected members of the KE family (whether or not some also experience comorbid disorders) would not have been possible without the detailed, careful phenotyping performed by Vargha-Khadem and colleagues. In this context, precise characterization of the behavioral phenotype drove both the neural and genetic discoveries by enabling them to generate precise, testable hypotheses about the locations of the neural differences in affected and unaffected family members and about the possible genetic source of those differences. Given that the DIVA model predicts that damage to other neural regions will be associated with signs that may be similar to or different from those shown by affected KE family members, equally deep phenotyping of speech performance in other NDDs is necessary if we are to test hypotheses about the links between behavior and neural substrates and between neural substrates and genetic differences. These behavioral investigations should be guided by current models of speech production, neural development, and gene expression.

The knowledge that deep speech phenotyping has revealed about the KE family, *FOXP2*, and CAS more generally underscores the importance of several lines of research. For example, as suggested by Vargha-Khadem and her colleagues (2005), further research into the nature of the linguistic and sequencing deficits in affected members of the KE family, and in individuals with CAS more generally, is needed in order to determine whether they are a consequence of the core deficit in speech and oral praxis or whether they represent separable comorbidities (c.f [42].). Longitudinal studies of children with CAS or other developmental speech disorders are especially important in this regard, as are imaging studies that can reveal structural and functional neural differences in these children. More precise characterization of the speech production deficits in children with CAS and in children with primary language impairment using kinematic methods may be especially revealing and enable us to identify similarities and differences between these two groups and, thus, provide a better understanding of how speech production and language ability are related. Similarly, research documenting developmental trajectories of speech development in children with other known and novel genetic disorders is sorely needed if we are to link the effects of those genes on neural circuitry and thus on behavior.

The investigations outlined above will only succeed if they are grounded in careful, accurate descriptions of speech production in children with NDDs. The protocol used to elicit speech from children with NDDs will depend on the information needed, and several options are available. Elsewhere, we have detailed methods for eliciting valid speech production data even from minimally verbal children [10, 14, 16]. To specifically diagnose CAS, Strand [87] recommends a protocol including at least a language sample (e.g., [4]), structural-functional oral exam (e.g., [84]), and a dynamic motor speech exam (e.g., [88]). Shriberg and his colleagues [75, 76] describe the Speech Disorders Classification System (SCDS), a detailed protocol including 15 speech tests and tasks that was developed specifically for the purpose of etiologically classifying pediatric speech sound disorders of unknown origin. However, a protocol including a 10- or 15-min spontaneous speech/language sample, a simple syllable repetition task that includes a variety of consonants and vowels from the child’s native language, and, for more able children, a standardized test of articulation (e.g., [31]) and a diadochokinetic task (e.g., “say ‘pataka’ as fast and as accurately as you can on one breath”) can provide enough information to identify the presence of CAS or childhood dysarthria when coded for signs of these disorders. Finally, an estimate of speech severity can be made using, for example, a visual analog scale rating [10, 14, 16].

The added value that these assessments offer to behavioral phenotyping in NDDs is that they will elucidate behavior that is specific to speech and that can be separated from language performance. Communicating by speech includes, but is not limited to, the ability to understand and produce language. Since producing speech also requires oromotor skill and the ability to use one’s own auditory feedback to refine pronunciation, deep speech phenotyping in NDDs can reveal how harnessing these skills for the purpose of communication is affected by genetic differences and shaped by the different behaviors and experiences that those differences engender. Documenting how speech development is affected (or not) and the existence (or lack) of speech disorders in different NDDs will increase our understanding of how the brain networks associated with communication are affected in NDDs. This will offer us deeper insights into the complex and interwoven interactions between genes, the brain, and behavior in speech, this most human of behaviors.

## Data Availability

N/A.

## Abbreviations

ASD: Autism spectrum disorder
ASHA: American Speech-Language-Hearing Association
CAS: Childhood apraxia of speech
CNV: Copy-number variation
IQ: Intelligence quotient
MSD-NOS: Motor speech disorder, not otherwise specified
NDD: Neurodevelopmental or genetic disorder
VBM: Voxel-based morphology
